# The Long Arc of Self-Rated Health Research

**DOI:** 10.1093/geroni/igaf122.081

**Published:** 2025-12-31

**Authors:** Ellen Idler, Ted Johnson

## Abstract

Research with older populations that asks respondents to assess their health with a single question began with the Duke Longitudinal Studies of Normal Aging in the 1950s. Early patterns that emerged were that physicians tended to rate overall health more negatively than study subjects, and that respondent “optimism” about health increased with age. In the 1980s a series of population-based studies of older respondents found that “poor” SRH was a significant predictor of all-cause mortality, even when physical health and other important covariates were well-characterized. Throughout the 1990s and 2000s literally hundreds of studies of representative, population-based samples in countries around the world have shown nearly universally that SRH is a reliable predictor of mortality, despite many forms of the question and different response categories. Since then, researchers have focused on understanding the precursors of these ratings; finding novel covariates that “explain” the effect and/or narrow it to a “true” self-rating of health; identifying sociodemographic groups for which the effect may be modified; and exploring the phenomenon of health optimism in old age. In this symposium, Quesnel-Vallée will present current research comparing SRH with algorithm-derived diagnoses. Jylhä will report on analyses of qualitative and quantitative data for a Finnish cohort aged 90+. Ferraro will present research on SRH and dual (physical and cognitive) functionality. Idler will present analyses of SRH and mortality where causes of death are disaggregated by treatability and preventability. The discussant will situate this current research in its long history and current translational applications.

